# Degradation of Bacterial Quorum Sensing Signaling Molecules by the Microscopic Yeast *Trichosporon loubieri* Isolated from Tropical Wetland Waters

**DOI:** 10.3390/s131012943

**Published:** 2013-09-25

**Authors:** Cheng-Siang Wong, Chong-Lek Koh, Choon-Kook Sam, Jian Woon Chen, Yee Meng Chong, Wai-Fong Yin, Kok-Gan Chan

**Affiliations:** 1 Division of Genetics and Molecular Biology, Institute of Biological Sciences, Faculty of Science, University of Malaya, Kuala Lumpur 50603, Malaysia; E-Mails: cswong2000@gmail.com (C.-S.W.); cjw246@hotmail.com (J.W.C.); cym_um@hotmail.com (Y.M.C.); yinwaifong@yahoo.com (W.-F.Y.); 2 Natural Sciences and Science Education AG, National Institute of Education, Nanyang Technological University, 1 Nanyang Walk, Singapore 637616, Singapore; E-Mails: chonglek.koh@nie.edu.sg (C.-L.K.); choonkook.sam@nie.edu.sg (C.-K.S.)

**Keywords:** basidiomycetous, biosensor, lactonase, *N*-acylhomoserine lactone, quorum sensing, quorum quenching, Rapid Resolution Liquid Chromatography, *Trichosporon loubieri*, yeast

## Abstract

Proteobacteria produce *N*-acylhomoserine lactones as signaling molecules, which will bind to their cognate receptor and activate quorum sensing-mediated phenotypes in a population-dependent manner. Although quorum sensing signaling molecules can be degraded by bacteria or fungi, there is no reported work on the degradation of such molecules by basidiomycetous yeast. By using a minimal growth medium containing *N*-3-oxohexanoylhomoserine lactone as the sole source of carbon, a wetland water sample from Malaysia was enriched for microbial strains that can degrade *N*-acylhomoserine lactones, and consequently, a basidiomycetous yeast strain WW1C was isolated. Morphological phenotype and molecular analyses confirmed that WW1C was a strain of *Trichosporon loubieri*. We showed that WW1C degraded AHLs with *N*-acyl side chains ranging from 4 to 10 carbons in length, with or without oxo group substitutions at the C3 position. Re-lactonisation bioassays revealed that WW1C degraded AHLs via a lactonase activity. To the best of our knowledge, this is the first report of degradation of *N*-acyl-homoserine lactones and utilization of *N*-3-oxohexanoylhomoserine as carbon and nitrogen source for growth by basidiomycetous yeast from tropical wetland water; and the degradation of bacterial quorum sensing molecules by an eukaryotic yeast.

## 1. Introduction

Many bacterial species modulate their gene expression as a function of their population density, a regulatory process known as quorum sensing (QS) [1–3]. While several distinct families of QS systems have been described, the most intensively studied QS signaling system relies on the production of *N*-acylhomoserine lactones (AHLs), used by diverse Gram negative bacteria [4,5]. All AHLs share an identical homoserine lactone ring, but differ in the *N*-linked acyl side chains, spanning from 4 to 18 carbons in length, with or without saturation or C3 hydroxy- or oxo- substitutions [6]. AHL-dependent QS regulates very diverse physiological functions [2,4,7], including several pathogenicity-related functions in plants and animal pathogens, such as *Erwinia carotovora* [8] and *Pseudomonas aeruginosa* [9]. Since AHL-mediated signaling mechanisms are widespread and highly conserved in many pathogenic bacteria, they were proposed as a novel target for the control of bacterial infectious diseases [10].

Evidence is accumulating that inhibition of QS, a mechanism known as quorum quenching (QQ), can be achieved through chemical inhibitors of the perception of QS signals. Some eukaryotes, including the macroalgae *Delisea pulchra* and *Flustra foliacea*, filamentous fungi, and several plants (*Pisum sativum*, *Myristica cinnamomea*) have been found to produce AHL antagonists [11–16]. Another strategy is the production of enzymes that degrade and inactivate the signaling molecules, as reported in a range of bacteria belonging to the Gram negative and Gram positive groups, in plants during germination, and in mammalian cells and forest root-associated fungi [17–19].

Since there is not much work done on the isolation of quorum quenching microorganisms from tropical marine waters, hence we have decided to choose this as our sampling site. In this paper, we report the surprising isolation of an AHL-degrading yeast, *Trichosporon loubieri* strain WW1C, from a sample of tropical wetland water collected in Malacca (Malaysia), by using a chemically defined medium. Strain WW1C could use *N*-3-oxohexanoylhomoserine (3-oxo-C6-HSL) as the sole carbon and nitrogen source, and degrade AHLs with acyl side chains ranging from 4 to 10 carbons in length, with or without 3-oxo substitutions.

## 2. Experimental Section

### 2.1. Yeast, Bacterial Strains and Culture Media

*T. loubieri* strain WW1C was isolated from wetland water collected in a sterile plastic container at subsurface level (5 cm beneath water level) in 2007 in Malacca (2°11′8″N, 102°15′2″E), Malaysia. *Escherichia coli* strain DH5α was used as a host for DNA manipulations. *Agrobacterium tumefaciens* strain NTL4 [pLZR4], *Chromobacterium violaceum* CV026 [20] and *E. coli* [pSB401] [21] were used as AHL biosensors to detect exogenous AHL. All bacterial strains were grown in Luria-Bertani (LB) medium (10 g/L NaCl, 10 g/L tryptone, and 5 g/L yeast extract). *T. loubieri* strain WW1C was initially enriched and selected in KGm medium and subsequently grown in LBm (same as LB, except for the use of 25 g/L NaCl). KGm medium was modified from KG medium [18]. It comprised a basal medium containing 1.25 g of NaCl, 0.75 g of KCl, 0.25 g of Na_2_SO_4_, 7.5 g of KH_2_PO_4_, 0.5 g of MgCl_2_, 0.25 g of CaCl_2_, 0.3 g of NH_4_Cl (unless otherwise stated), and 1.0 g of 2-(*N*-morpholino)-ethanesulfonic acid (MES), in 1 L of sterile distilled water. After this basal medium was autoclaved and cooled, filter-sterilized (0.22 μm pore size) FeCl_3_, MnCl_2_, and ZnCl_2_ solutions were added to it to final concentrations 5 mg/L, 2.5 mg/L, and 0.6 g/L, respectively. 3-oxo-C6-HSL was used as the sole carbon source (final concentration 50 μg/mL). All LB and LBm media used in this study were buffered with 50 mM 3-(*N*-morpholino) propanesulfonic acid (MOPS) to pH 5.5 to prevent spontaneous degradation of AHLs [22]. For growth of *E. coli* [pSB401], growth media were supplemented with tetracycline (5 mg/L) and solidified with bacto-agar (15 g/L).

### 2.2. Enrichment and Isolation Procedures

The enrichment and isolation procedures were as described by Chan *et al.* [17] with slight modification. One milliliter of Malacca wetland water was added to 3 mL of KGm medium and supplemented with 3-oxo-C6-HSL (final concentration 50 μg/mL) as sole carbon source. This mixture was incubated at 28 °C with shaking (220 rpm). After 48 h, 150 μL of the suspension was inoculated into 3 mL (5% v/v) of fresh KGm medium containing 3-oxo-C6-HSL (50 μg/mL). The same procedure was repeated until the fifth enrichment cycle when a diluted suspension was plated onto LBm agar and 3-oxo-C6-HSL-containing KGm agar to isolate individual colonies.

### 2.3. Strain Identification

The genomic DNA of *T. loubieri* strain WW1C was isolated from an overnight culture as described by Sambrook *et al.* [23]. The internal transcribed spacer (ITS) region and 18S rDNA gene were amplified from the genomic DNA by using universal primer sets ITS1/ITS4 and NS1/NS8, respectively [24]. The following PCR conditions were used: initial denaturation at 94 °C for 3 min, followed by 30 cycles of 30 s of denaturation at 94 °C, 30 s of annealing (50 °C for ITS and 55 °C for 18S rDNA), and 1.5 min of extension at 72 °C, and then a final extension at 72 °C for 10 min. Ligation, transformation, DNA sequencing, and phylogenetic analysis were as previously described [17].

### 2.4. Growth Studies

An overnight culture of WW1C (20 mL) was washed four times in successively smaller volumes (10, 5, 2.5, and 1 mL) of PBS (100 mM, pH 6.5) and the pelleted cells were resuspended in 500 μL of PBS. Next, 100 μL aliquots of the suspension were separately inoculated into 100 mL of KGm medium containing 3-oxo-C6-HSL (final concentration 50 μg/mL) and ammonium chloride-depleted KGm medium containing 3-oxo-C6-HSL (final concentration 50 μg/mL). Incubations were performed at 28 °C with shaking (220 rpm). OD_600_ was determined at 2 h intervals over a 24 h period. Doubling times were estimated from the linear portion of the growth curves obtained. Statistical analysis (two-tailed paired t-test) was done using Microsoft Excel.

### 2.5. In Vitro AHL Inactivation Assays with Resting Cells of WW1C

AHLs with acyl chains ranging from 4 to 14 carbons were purchased from Sigma-Aldrich or received as gifts from Professor Paul Williams (Nottingham, UK). They are: *N*-butanoylhomoserine lactone (C4-HSL), *N*-hexanoylhomoserine lactone (C6-HSL), *N*-heptanoylhomoserine lactone (C7-HSL), *N*-octanoylhomoserine lactone (C8-HSL), *N*-decanoylhomoserine lactone (C10-HSL), *N*-dodecanoylhomoserine lactone (C12-HSL), *N*-tetradecanoylhomoserine lactone (C14-HSL), *N*-(3-oxohexanoyl)homoserine lactone (3-oxo-C6-HSL), *N*-(3-oxooctanoyl)homoserine lactone (3-oxo-C8-HSL), *N*-(3-oxododecanoyl)homoserine lactone (3-oxo-C12-HSL), and *N*-(3-oxotetra- decanoyl)homoserine lactone (3-oxo-C14-HSL).

AHL degradation by WW1C resting cells was assessed as described by Chan *et al.* [25]. WW1C cells (100 mL, normalized to an OD_600_ of 1.0) grown in liquid LBm for 48 h were harvested by centrifugation, washed and resuspended in 1 mL of PBS (100 mM, pH 6.5). The resting WW1C cell suspension was used to rehydrate air-dried AHL aliquots to a final concentration 0.5 mg/mL. The mixtures were incubated at 28 °C for 24 h with shaking (220 rpm). The reactions were stopped at appropriate intervals by addition of ethyl acetate or boiling [25]. Ethyl acetate containing the residual AHL molecules was removed and evaporated to dryness before reconstituted in an appropriate volume of acetonitrile. Residual AHL molecules were detected using the AHL biosensor *C. violaceum* CV026 and lux-based biosensor *E. coli* [pSB401]. *E. coli* strain DH5α and PBS (100 mM, pH 6.5) were used as negative controls. The method of Yates *et al.* [22] was used to detect a possible lactonase activity that opened the lactone ring of C6-HSL. The method is based on recyclization of the opened lactone ring of hydrolyzed C6-HSL under acidic conditions (pH < 2) and detection of the reformed C6-HSL by using *C. violaceum* CV026. Acidification of the reaction mixture to pH 2 was achieved with 100 mM HCl. To ascertain whether strain WW1C degradation of AHL was based on a heat sensitive enzymatic reaction, we boiled the strain WW1C cells in 100 °C for 10 min, and ensured no viable cells remained by plating on rich medium. We analysed strain WW1C degradation on C6-HSL after 0- and 24-h incubation with boiled and resting cells of *T. loubieri* strain WW1C (as control). Residual C6-HSL was detected by using the bioluminescent sensor strain *E. coli* [pSB401].

### 2.6. Rapid Resolution Liquid Chromatography (RRLC) Analysis of AHL Degradation

AHL concentration was determined using Reverse-phase Rapid Resolution Liquid Chromatography (RRLC, Agilent Inc., Santa Clara, CA, USA). Samples were withdrawn at 0, 15, 60 and 120 min. RRLC analysis of AHLs (5 μg) was performed on an Agilent ZORBAX Eclipse XDB-C18 column (4.6 × 50 mm, 1.8 μm particle size) using an Agilent Technologies 1200 series Rapid Resolution LC system and eluting with an acetonitrile-water (35:65, v/v) isocratic profile for 3 min at a constant flow rate of 0.7 mL/min and monitored at 210 nm. Both the retention time and spectral properties of the peaks were compared with those of synthetic AHL standards. To estimate the amount of residual AHLs, the extracted AHLs from the AHL inactivation assay were analyzed using RRLC as previously described [17].

### 2.7. Nucleotide Sequence Accession Numbers

The ITS region and 18S rDNA gene sequences of *T. loubieri* strain WW1C were deposited at GenBank under the GenBank accession numbers FJ383170 and FJ426276, respectively. All other sequences were from the GenBank database. GenBank accession numbers: *Trichosporon loubieri* (AB001759), *Trichosporon dulcitum* (AB001755), *Trichosporon gracile* (AB001756), *Trichosporon multisporum* (AB001764), *Cryptococcus fragicola* (AB035588), *Trichosporon ovoides* (AB001765), *Bullera formosensis* (AB072235), *Kockovaella calophylli* (AB042222), *Trichosporon loubieri* strain ATCC MYA-26 (AY101607), T*richosporon loubieri* strain CBS 7065 (AF444438), *Trichosporon mycotoxinivorans* (AJ601389), *Trichosporon multisporum* strain CBS2495 (AF414695), *Trichosporon gracile* strain CBS 8519 (AF444456), *Trichosporonales* sp. LM117 (EF060484), *Trichosporon otae* (AB180196), *Cryptococcus laurentii* strain WM 601 (EF568051), *Cryptococcus curvatus* strain ATCC 10567 (EU266558).

## 3. Results and Discussion

### 3.1. Enrichment and Isolation of Microbial Isolates

KGm medium was used in this study. We have previously used KG medium to isolate QQ bacteria including *Acinetobacter* sp., *Burkholderia* sp., *Klebsiella* sp. [25]; and a bacterium previously considered as unculturable [17]. The growth medium became turbid within 48 h after inoculation with Malacca wetland water. Subsequently, bacterial culture was plated onto both LBm agar and 3-oxo-C6-HSL-containing KGm agar. Pure cultures were obtained after several successive streaks from randomly selected single colonies. One isolate, designated as strain WW1C, was selected for further analysis.

### 3.2. Colony and Cell Morphology

**Figure 1.**
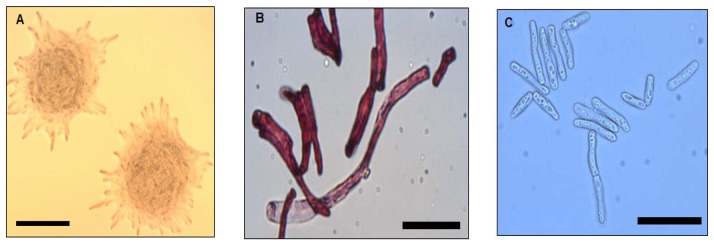
Colonial and cell morphology of strain WW1C. (**A**) Photograph of colonies grown on LBm; (**B**) Light micrograph of Gram-stained cells of strain WW1C grown on LBm plates; (**C**) Light micrograph of a droplet of overnight LBm broth culture of strain WW1C stained with crystal violet. The bars represent 500 μm (A), 50 μm (B), and 100 μm (C).

Strain WW1C grown on LBm plates for 48 h at 28 °C yielded white, opaque, domed-shaped colonies with colony diameters of 5 mm. Colonies were essentially round, with filamentous edges and appeared dry and powdery (Figure 1A). The wrinkled appearance became more prominent with prolonged incubation (>48 h). Well-developed hyphal structures and barrel-shaped arthroconidia were observed (Figure 1B). Under a light microscope, pleomorphic yeast-like cells with cell length ranging from approximately 30 to 150 μm were observed (Figure 1C). Based on morphological features, strain WW1C was provisionally considered to be of a member of the kingdom Fungi.

### 3.3. Confirmation of Strain WW1C as Trichosporon loubieri

**Figure 2.**
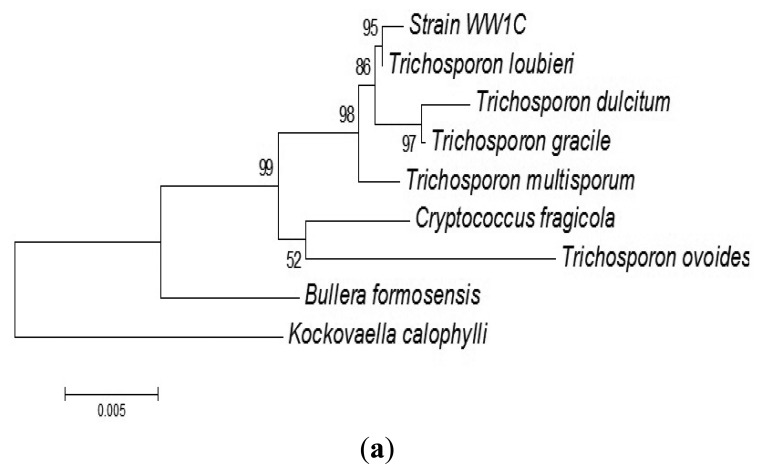

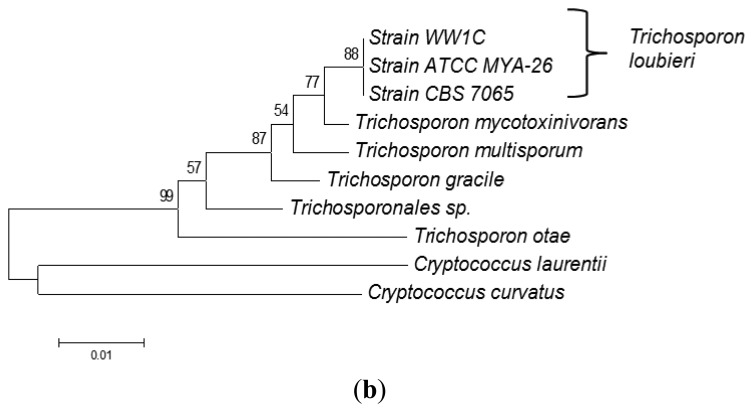
(**a**) 18S rDNA gene-based phylogenetic tree showing the phylogenetic position of strain WW1C generated using Neighbour-Joining algorithm. The horizontal bar at the bottom represents evolutionary distance as 0.005 changes per nucleotide position, determined by measuring the lengths of the horizontal lines connecting the species. The numbers (bootstrap values as percentages of 1,000 replications) provide support for the robustness of the adjacent nodes. *Kockovaella calophylli* was used as an outgroup; (**b**) ITS region-based phylogenetic tree showing the phylogenetic position of strain WW1C generated using Neighbour-Joining algorithm. The horizontal bar at the bottom represents evolutionary distance as 0.01 changes per nucleotide position, determined by measuring the lengths of the horizontal lines connecting the species. The numbers (bootstrap values as percentages of 1,000 replications) provide support for the robustness of the adjacent nodes. *Cryptococcus laurentii* and *Cryptococcus curvatus* were used as an outgroup.

The complete sequences of the ITS region and 18S rDNA of WW1C were determined. The 18S rDNA gene sequence of WW1C shared 99% sequence identity with the 18S rDNA gene of *Trichosporon loubieri*. A further phylogenetic analysis based on its 18S rDNA gene (Figure 2a) and ITS nucleotide sequences (Figure 2b) supported the conclusion that WW1C is a strain of *T. loubieri*. Strain WW1C grew well at 37 and 42 °C in LBm broth supplemented with 0.01 μg/mL cycloheximide (data not shown), two typical physiological characteristics that distinguish *T. loubieri* from other species of *Trichosporon* [26]. Hence, the AHL-metabolizing isolate WW1C was named *T. loubieri* strain WW1C.

### 3.4. T. loubieri Strain WW1C Used 3-oxo-C6-HSL as Energy and Nitrogen Sources for Growth

**Figure 3.**
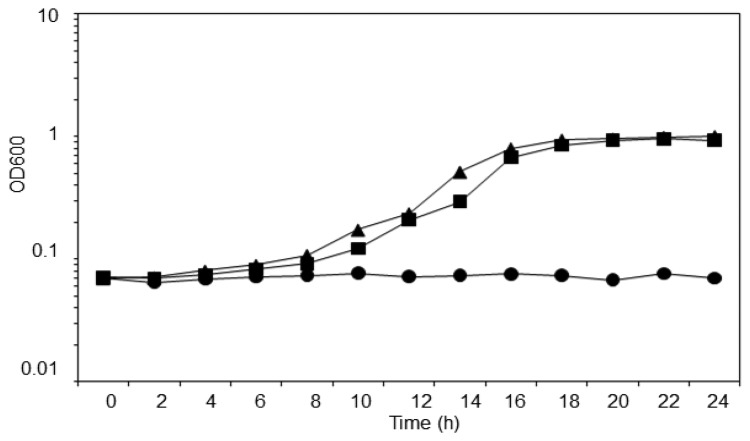
Growth of *T. loubieri* strain WW1C on with 3-oxo-C6-HSL as the sole energy source. Cell growth was monitored at OD_600_ (on semi-log scale), determined over 24 h in ammonium chloride-replete (triangles) and depleted (squares) minimal medium supplemented with 3-oxo-C6-HSL. Residual growth in minimal medium without any carbon or nitrogen source is also shown (circles).

*T. loubieri* strain WW1C was isolated based on its ability to use 3-oxo-C6-HSL as a sole carbon and energy source. The strain was able to grow in KGm medium (Figure 3) containing 3-oxo-C6-HSL (50 μg/mL), with a doubling time of approximately 2.5 h. WW1C also grew well in ammonium chloride-depleted KGm medium containing 3-oxo-C6-HSL (50 μg/mL), confirming that it could use 3-oxo-C6-HSL as carbon and nitrogen source. No statistically significant difference was detected between the two growth curves using a two-tailed paired t-test (t = 3.0192, df = 12, *p* = 0.01). No growth was observed in KGm medium lacking 3-oxo-C6-HSL (Figure 3). *Trichosporon* species have been readily isolated from various environments and they are capable of degrading aromatic compounds. For example, *Trichosporon cutaneum* is capable of growing on phenol and resorcinol as the carbon source [27].

### 3.5. Substrate Specificity of T. loubieri Strain WW1C

**Figure 4.**
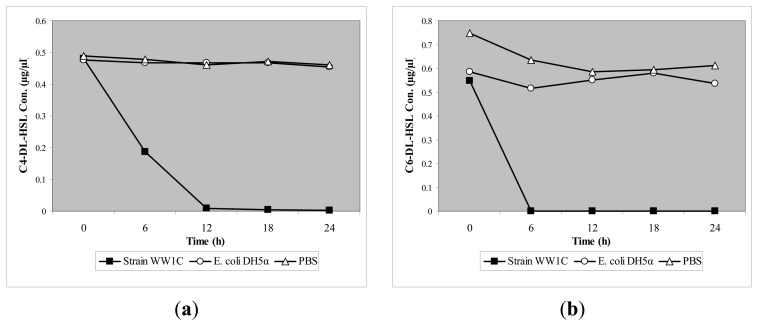

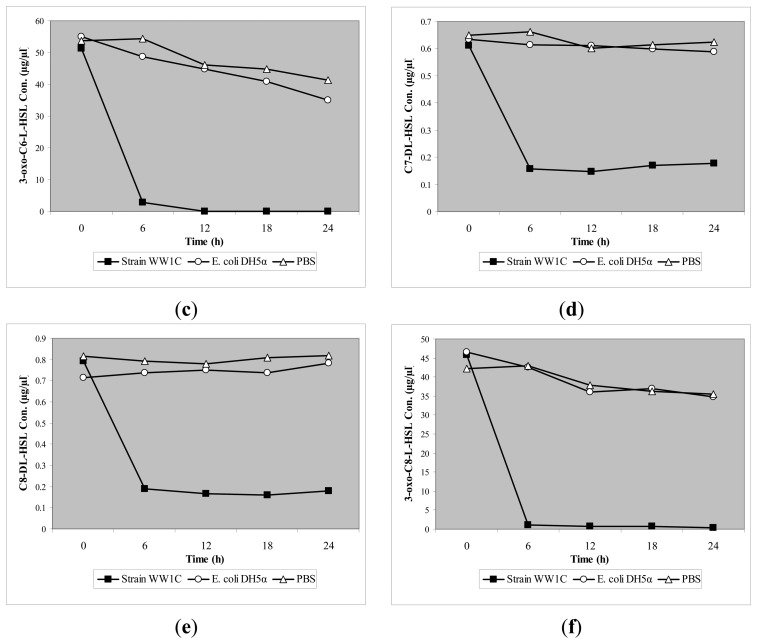
RRLC analysis of AHLs degradation after 0-, 6-, 12-, 18-, and 24-h incubation with T. loubieri strain WW1C.Degradation of (**a**) C4-HSL; (**b**) C6-HSL; (**c**) 3-oxo-C6-HSL; (**d**) C7-HSL; (**e)** C8-HSL and (**f**) 3-oxo-C8-HSL by *T. loubieri* strain WW1C (square). Residual AHLs as measured based on calibration curve derived from calibration standards ranging from 0.0025 to 0.15 μg/μL. *E. coli* DH5α (circle) and incubation buffer (PBS, 100 mM, pH 6.5) (open triangle) served as negative controls. AHLs were incubated with *T. loubieri* strain WW1C cells, *E. coli* DH5α and PBS for 24 h, and extracted at 0-, 6-, 12-, 18- and 24-h, and analysed by RRLC.

**Figure 5.**
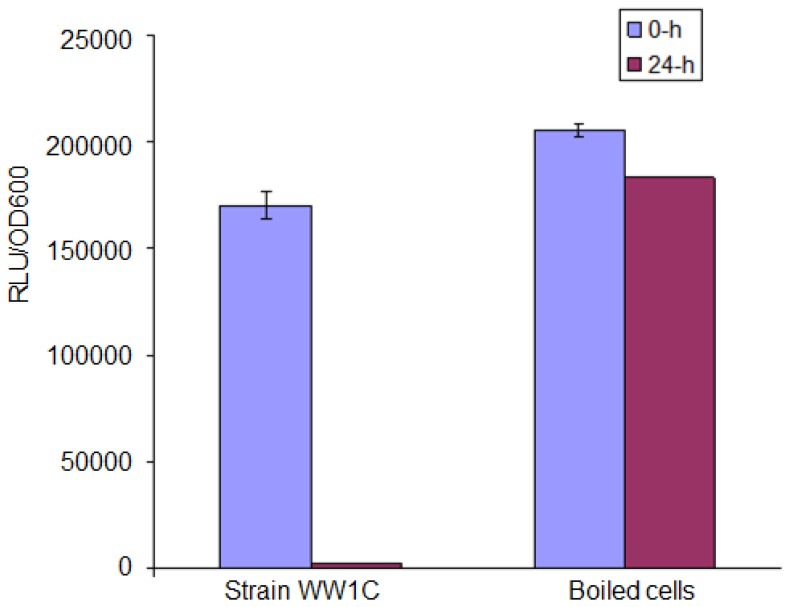
Analysis of C6-HSL after 0- and 24-h incubation with boiled and resting cells of *T. loubieri* strain WW1C. C6-HSL was incubated with boiled and resting cells of *T. loubieri* strain WW1C. Samples were withdrawn at 0 h (blue) and 24-h (purple), residual C6-HSL was detected by using the bioluminescent sensor strain *E. coli* (pSB401).

**Figure 6.**
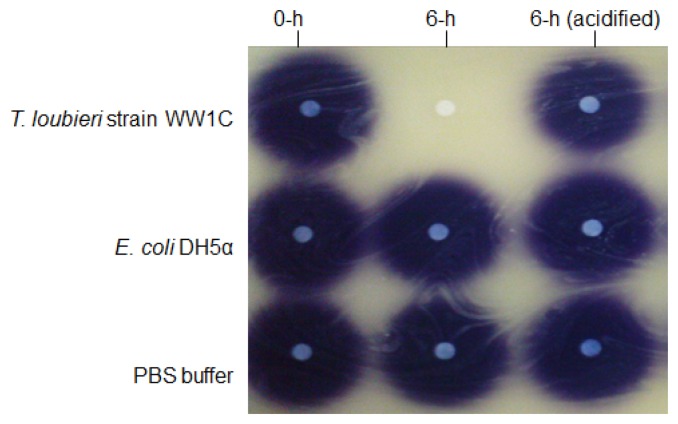
Identification of degradation products of C6-HSL incubated with *T. loubieri* strain WW1C. C6-HSL incubated with *T. loubieri* strain WW1C cells for 0 h (0-h), 6 h (6-h), and samples withdrawn at 6 h and acidified (6-h acidified) to promote re-lactonisation. Bacterial names were labeled on the left of the figure. *E. coli* DH5α and extraction buffer (PBS 100 mM, pH 6.5) were used as the negative controls. Residual C6-HSL was detected by using CV026 and visualized as purple spot.

*T. loubieri* strain WW1C effectively degraded C4-HSL (Figure 4a), C6-HSL (Figure 4b), 3-oxo-C6-HSL (Figure 4c), C7-HSL (Figure 4d), C8-HSL (Figure 4e) and 3-oxo-C8-HSL (Figure 4f). Thus, although *T. loubieri* strain WW1C was initially isolated based on its ability to degrade 3-oxo-C6-HSL, it was subsequently found to efficiently degrade a variety of AHLs with carbon chains ranging in length from C4 to C10 and substitutions at the C3 position (Table 1). The most efficiently degraded molecule was C8-HSL, with an estimated activity of 4.04 μg AHL/h/(10^5^ CFU). Remarkably, strain WW1C also degraded C7-HSL, an unsubstituted AHL with an odd number *N*-acyl side chain which is not commonly produced by proteobacteria [25]. No significant degradation of C10-HSL, C12-HSL, C14-HSL 3-oxo-C12-HSL, and 3-oxo-C14-HSL was detected (data not shown). In all AHL degradation assays, no degradation of AHLs was detected in the negative controls involving *E. coli* DH5α and PBS buffer (100 mM, pH 6.5) (data not shown). Boiled resting WW1C cells failed to degrade C6-HSL (Figure 5), suggesting that strain WW1C's AHL degradation occurred by an enzymatic inactivation mechanism that might involve a heat-sensitive lactonase. To confirm this fact, and following the method of Yates *et al.* [22], we performed a relactonisation and the acidified reaction mixture derived from incubation of C6-HSL with resting *T. loubieri* strain WW1C cells was able to induce violacein production in *C. violaceum* CV026 (Figure 6), indicating that recyclization of the opened lactone ring occurred under acidic conditions and verifying that strain WW1C indeed degraded AHLs using a lactonase enzyme.

The degradation of AHLs by *T. loubieri* strain WW1C expands the list of diverse metabolic traits exhibited by members of this genus. To date, QQ activity by fungi has only been reported for *Penicillium* spp. [14] and a number of forest root-associated fungi belonging to the *Ascomycota* and *Basidiomycota* lineages [19]. However, it is noteworthy to distinguish the difference between the quorum quenching mechanisms in *Penicillium* spp. and the *T. loubieri* strain WW1C because the former produce biomimic compounds named penicillic acid and patulin [14], but strain WW1C used enzyme(s) to degrade AHLs. To the best of our knowledge, this is the first report of a basidiomycetous yeast capable of degrading a variety of AHLs and growing on 3-oxo-C6-HSL as carbon and nitrogen source. It has been commonly found that QQ bacteria are abundantly found in natural habitats [28–34] and much work has been focused on these bacterial QQ mechanisms [35]. Our discovery of the QQ *T. loubieri* strain WW1C identifies a new member of the known QQ microorganisms which has not been previously reported.

Although *T. loubieri* strain WW1C was initially isolated based on its ability to degrade 3-oxo-C6-HSL, it was subsequently found to efficiently degrade a variety of AHLs with a carbon chain ranging from C4 to C10. It degraded C8-HSL most efficiently. Remarkably, strain WW1C also degraded C7-HSL, an unsubstituted AHL with an odd number *N*-acyl side chain. Boiled resting WW1C cells failed to degrade C6-HSL, suggesting that strain WW1C may possess a heat-sensitive lactonase activity. The results of the method of Yates *et al.* [22] confirmed that the degradation of AHLs in *T. loubieri* strain WW1C is by an AHL lactonase activity. The presence of a putative aiiA homologue was investigated in WW1C using the PCR method. However, no amplicon was observed (data not shown). It is speculated that WW1C may possess a novel AHL lactonase distinct from the aiiA-class of AHL lactonase. Further study is required to determine the gene involved in the metabolic pathway and the metabolic fate of the AHLs.

Recently, it has become apparent that like bacteria, fungi also utilize QS to regulate population-level behaviors such as pathogenesis [36]. Various fungal signaling molecules have been identified, e.g., farnesol and tyrosol in *Candida albicans* and trisporic acid produced by zygomycetes [36]. We also examined AHL production by *T. loubieri* strain WW1C by using both AHL biosensors (*A. tumefaciens* NTL4 (pLZR4) and CV026) and LC-MS analysis. When tested under a range of incubation temperatures (16 °C, 23 °C, and 28 °C) and high salinity (2.5% w/v NaCl), no production of AHL was detected in WW1C (data not shown).

The discovery of the AHL degrading activity in *T. loubieri* strain WW1C raises the question of the purpose of the existence of such catabolic activity in eukaryotic microorganisms although QQ properties have been widely documented. In the soil, some bacteria are known to have chitinolytic, antibiosis or antifungal activities regulated by QS [37]. For example, phenazine antibiotics produced by *Pseudomonas aureofaciens* have been shown to protect wheat from take-all, a disease caused by the fungus *Gaeumannomyces graminis* var. *tritici* [38,39]. The degradation of AHL could be a strategy developed by the fungi to disrupt the QS system and the deleterious functions of bacteria, thereby giving the fungi survival advantages over QS bacteria in the competitive habitats. Evidence from a number of reports has suggested that higher eukaryotes use QQ mechanisms to control colonizing or pathogenic bacteria.

## 4. Conclusions and Outlook

In this study, the *T. loubieri* strain WW1C degraded AHL signaling molecules and furthermore metabolized them as energy sources. It is tempting to speculate that yeasts may be able to sense and inactivate the bacterial QS signal molecules and metabolize them for cellular assimilation for growth. Very little is known about eukaryotic QQ systems as compared with bacterial QQ ones. Many intriguing questions arise from the identification of this novel QQ *T. loubieri* strain WW1C: what is the ecological role of the AHL-degrading mechanism in the environment? Could microscopic yeasts in the environment merely act as detritivores that degrade AHLs as one of their organic food sources? Is it possible that eukaryotic yeasts respond to AHLs and inactivate them in order to prevent QS-mediated phenotypes that may confer ecological advantage to QS bacteria to their detriment? Future work should be done to address the ecological role of this QQ yeast in the marine habitat in order to investigate whether QQ properties allow the microscopic eukaryotes (such as *T. loubieri* strain WW1C) to compete with QS proteobacteria.

## Figures and Tables

**Table 1.** Degradation of various AHLs by *T. loubieri* strain WW1C.

**Type of AHL**	Estimated Activity*
C4-HSL	1.23
C6-HSL	3.67
C7-HSL	3.04
C8-HSL	4.04
C10-HSL	0.24
C12-HSL	0
C14-HSL	0
3-oxo-C6-HSL	3.25
3-oxo-C8-HSL	2.99
3-oxo-C10-HSL	0.76
3-oxo-C12-HSL	0
3-oxo-C14-HSL	0

* Estimated activity is given as μg of AHL degraded/h/(10^5^ CFU). See text for AHL abbreviations.
